# Computational Design of Protein-Based Inhibitors of *Plasmodium vivax* Subtilisin-Like 1 Protease

**DOI:** 10.1371/journal.pone.0109269

**Published:** 2014-10-24

**Authors:** Giacomo Bastianelli, Anthony Bouillon, Christophe Nguyen, Dung Le-Nguyen, Michael Nilges, Jean-Christophe Barale

**Affiliations:** 1 Institut Pasteur, Unité de Bioinformatique Structurale, Département de Biologie Structurale et Chimie, Paris, France; 2 CNRS UMR 3528, Paris, France; 3 Institut Pasteur, Unité d’Immunologie Moléculaires des Parasites, Département de Parasitologie et de Mycologie & CNRS URA 2581, Paris, France; 4 CNRS, URA2581, Paris, France; 5 SYSDIAG, CNRS UMR3145 CNRS-BioRad, Montpellier, France; Agency for Science, Technology and Research - Singapore Immunology Network, Singapore

## Abstract

**Background:**

Malaria remains a major global health concern. The development of novel therapeutic strategies is critical to overcome the selection of multiresistant parasites. The subtilisin-like protease (SUB1) involved in the egress of daughter *Plasmodium* parasites from infected erythrocytes and in their subsequent invasion into fresh erythrocytes has emerged as an interesting new drug target.

**Findings:**

Using a computational approach based on homology modeling, protein–protein docking and mutation scoring, we designed protein–based inhibitors of *Plasmodium vivax* SUB1 (PvSUB1) and experimentally evaluated their inhibitory activity. The small peptidic trypsin inhibitor EETI-II was used as scaffold. We mutated residues at specific positions (P4 and P1) and calculated the change in free-energy of binding with PvSUB1. In agreement with our predictions, we identified a mutant of EETI-II (EETI-II-P4LP1W) with a *Ki* in the medium micromolar range.

**Conclusions:**

Despite the challenges related to the lack of an experimental structure of PvSUB1, the computational protocol we developed in this study led to the design of protein-based inhibitors of PvSUB1. The approach we describe in this paper, together with other examples, demonstrates the capabilities of computational procedures to accelerate and guide the design of novel proteins with interesting therapeutic applications.

## Introduction

With more than 400 millions infections worldwide, malaria remains a major public health issue, principally in sub-Saharan Africa. An effective vaccine would help reduce disease burden, but the best candidates are still in development or evaluation phase [1], [2]. The rapid development of multidrug-resistant *Plasmodium* [3] parasites necessitates accelerating the discovery of novel anti-malarial compounds to meet the needs of the agenda for malaria control and eradication [4].

In humans, *Plasmodium sp.* development comprises different stages, with the asexual intra–erythrocytic forms being responsible for the symptoms of the disease, such as fever, anemia, and cerebral malaria that can lead to death [5]. The erythrocyte invasion by *Plasmodium* merozoites critically depends on protease activities involved in both the daughter parasites egress from erythrocytes, and invasion into another erythrocyte. The parasite subtilisin-like protein 1 (SUB1) plays a critical role during both the hepatic and erythrocytic phases of *Plasmodium* biological cycle and is hence considered an interesting multi-stage target for developing a new class of anti–malarials [6] [7].

Most of the ancient therapies against *Plasmodium* are based on small molecules such as chloroquine, quinolones, antifolate, artemisinin derivatives, or atovaquone. The development of new classes of active molecules such as protein–based drugs or peptidomimetics [8], [9] is an active and promising field of research. Among protein–based drugs, dermaseptin S4 (DS4) was shown to irreversibly inhibit the *in* *vitro* parasite growth through a cytotoxic hemolytic activity. Dermaseptin S3 acts in a similar manner as DS4 but did not present hemolytic activity through a cytotoxic hemolytic activity [10].

In the design of protein–based drugs, most approaches use combinatorial libraries based on different screening methods such as phage [11], ribosome [12] or mRNA display [13]. Their use is wide–spread, in particular for selecting high-affinity protein binders, despite their limitations due to the library size and the large quantities of the target protein needed to perform screening. Moreover, when the selection is not based on binding but on inhibiting a crucial enzyme of the biological cycle, a rather complex selection system has to be employed. Computational protein design can be used to reduce the sequence/structure space that needs to be explored and thus accelerate the process of screening and selection of target inhibitors.

Here, we present a strategy for the computational design of protein-based inhibitors targeting the subtilisin–like 1 protease of the human parasite *Plasmodium vivax* (PvSUB1). PvSUB1 can be expressed as a recombinant active enzyme [14] [15], and a specific enzymatic assay allows one to evaluate specific inhibitors. To search for potential inhibitors of PvSUB1, we used a computational design strategy, employing as scaffold the small protein EETI-II (*Ecballium elaterium* trypsin inhibitor II) [16], a trypsin inhibitor extracted from *Ecballium elaterium*. The family of cystein–knot proteins, to which EETI-II belongs, and in particular the cyclotides [17], possesses interesting biochemical properties [18]. EETI-II is composed of 28 amino-acids and its three-dimensional structure is tightly constrained by 3 disulphide bridges that contribute to its rigidity and biological stability [19]. We opted for this scaffold because several studies showed the possibility to engineer this protein to obtain specific mutants [20], *via* the extension of the EETI bioactive loop [21] or by changing its sequence to change its specificity towards the targeted enzyme [22] [23] [24] [25].

Compared to studies using an iterative computational design procedure focused on electrostatic binding contributions and single mutants [26], or on re–designing a scaffold protein to bind to a specified region on a target protein [27], we here faced the additional challenge that the 3D structure of the target itself or a close sequence homologue was not known. Nonetheless, the use of state–of–the–art structure prediction, docking and scoring methods allowed us to successfully identify mutants of the scaffold EETI-II that inhibited the target PvSUB1 enzyme.

## Results and Discussion

**Figure 1:**
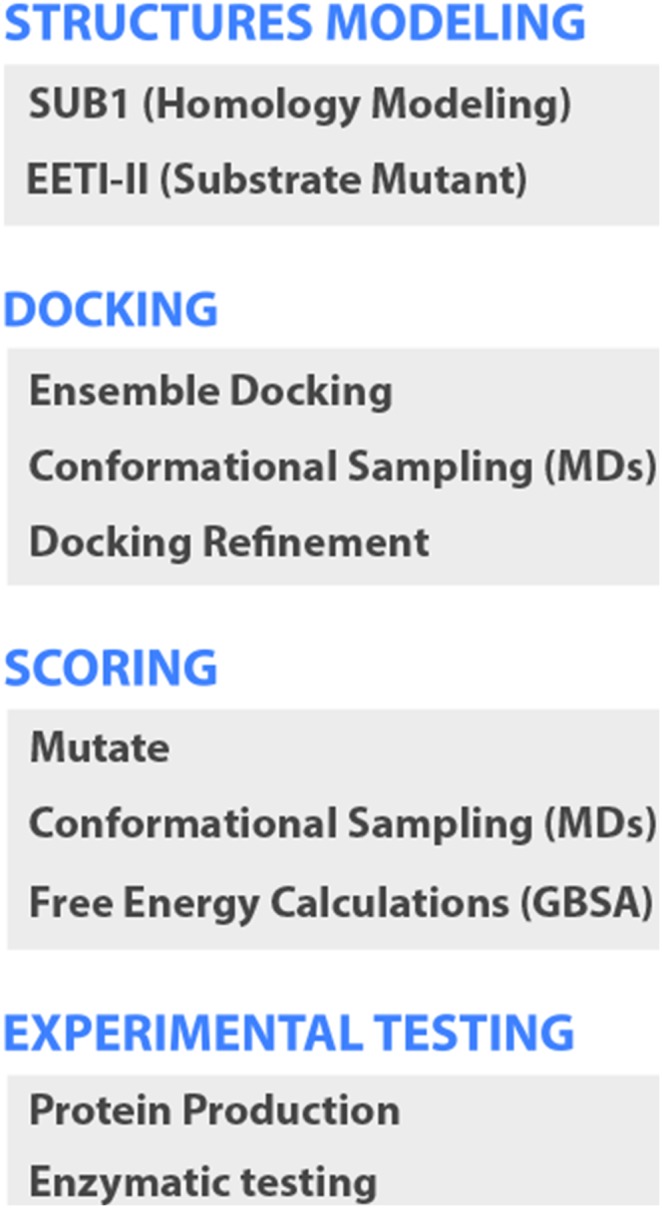
Computational Protein Design Strategy. Step 1: Prediction of the structure of the enzyme (PvSUB1) by comparative modelling and of the scaffold for mutational analysis (EETI-II-sub) by replacing one of the loops with a substrate sequence. Step 2: docking of EETI-II-sub to the target protein by ensemble docking procedure with several conformations from molecular dynamics simulations for each protein partner, and refinement of the best solutions. Step 3: mutation of the scaffold, conformational sampling and scoring of the mutants. Step 4: experimental testing by an enzymatic inhibitory assay on the recombinant enzyme of PvSUB1.

The computational protein design approach involved four steps (see Figure 1). The first step was the modeling of the structure of the enzyme (PvSUB1) and the scaffold (EETI-II). Because of the lack of an experimental PvSUB1 structure, we built structures based on sequence homology. We also generated the model of a mutant of EETI-II containing the substrate sequence of PvSUB1, which we called EETI-II- sub. The second step was the docking of EETI-II-sub to the target protein. We employed an ensemble docking procedure with several conformations obtained from molecular dynamics (MD) simulations for each protein partner to implicitly include flexibility in the docking, and refined the best docking solutions by molecular dynamics to obtain high-quality structures of the complex. The third step aimed at identifying mutants of EETI-II-sub that had higher binding affinity towards PvSUB1. In this step, we mutated residues in EETI-II-sub at the protein–protein interface of the complex, ran conformational sampling of the mutant with molecular dynamics, and calculated the free energy of binding via implicit solvent models based on the Generalized Born approximation (GBSA). The last step consisted in the experimental testing of the inhibitor by an enzymatic inhibitory assay specific for the PvSUB1 recombinant enzyme.

### Modeling and molecular dynamics simulations of PvSUB1

**Figure 2:**
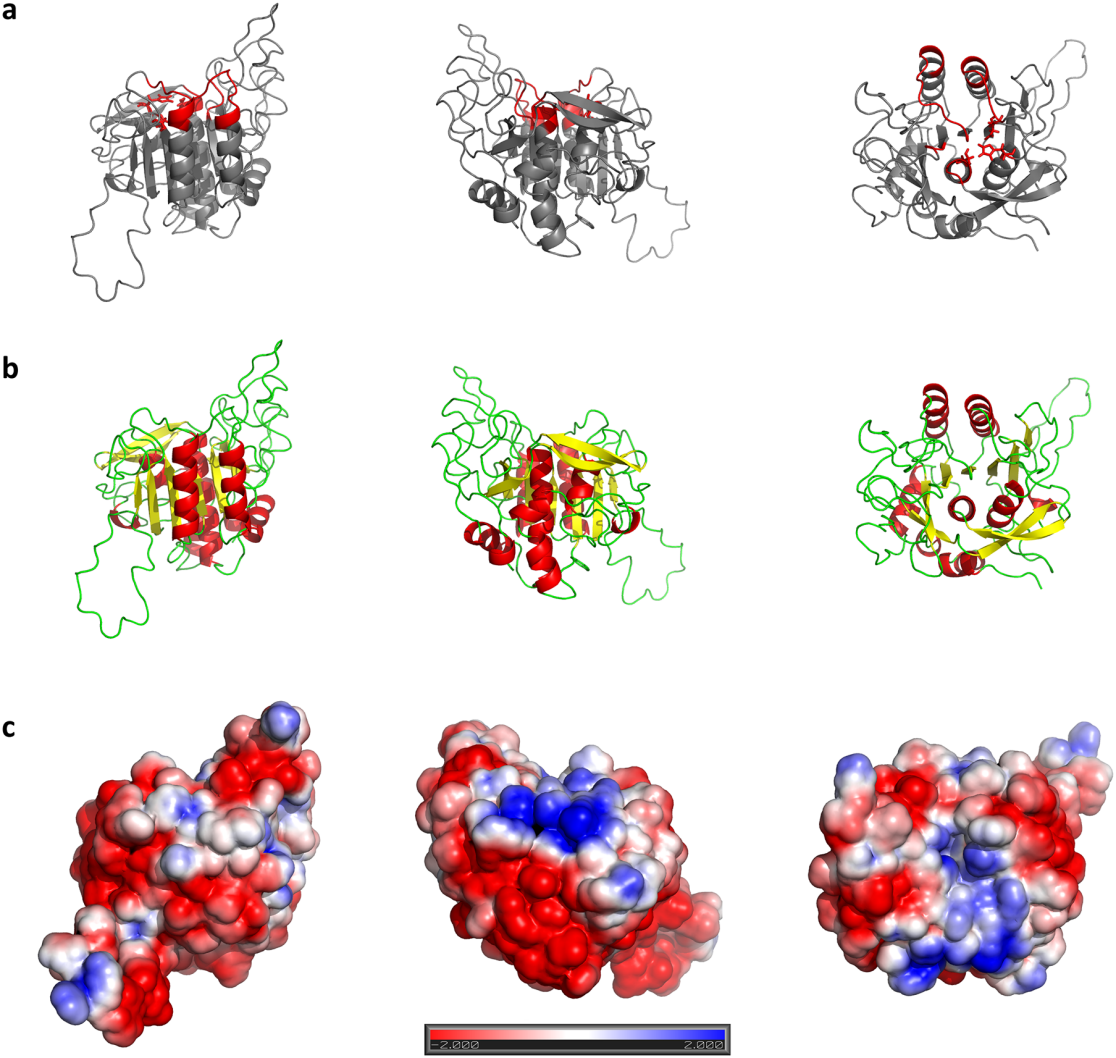
3D model of PvSUB1 catalytic domain. **A:** Highlighted in red is the region forming the substrate binding pocket and red sticks correspond to the residues that form the catalytic triad; **B:** Cartoon representation of secondary structures; **C:** APBS surface electrostatic representation.

In order to generate a reliable 3D-model of PvSUB1, we used the procedure described in our previous publication where we modeled the structure of PfSUB1, a close homologous of PvSUB1 [14]. A similar homology modeling strategy generated 3D-models of PfSUB1 used to identify small-molecule inhibitors of PfSUB1 with an *in* *silico* screening approach [15]. The particular challenge of obtaining a high quality 3D model of PvSUB1 was the low sequence identity with the available templates (only ∼30%, just above the “twilight zone” for homology modeling). Using state of the art modeling methods, it is possible to generate homology models with a Cα RMSD<1.0 Å when the sequence identity with the template is >50%. With sequence identity below 25%, larger divergences from the target structure can appear, making the model less precise [28]. However, our previous analysis had shown that major divergences between the PvSUB1 sequence and the structural templates were localized in regions distant from the catalytic groove that binds the substrate, and that the sequence identity in the substrate binding area is *>*30% [14], [15]. In addition, to evaluate the stability and the overall quality of the PvSUB1 model (Figure 2), we performed multiple molecular dynamics simulations of our model PvSUB1 and of two of the templates used in the modeling, subtilisins BPN (1TO2) and Carlsberg (1R0R).

**Figure 3:**
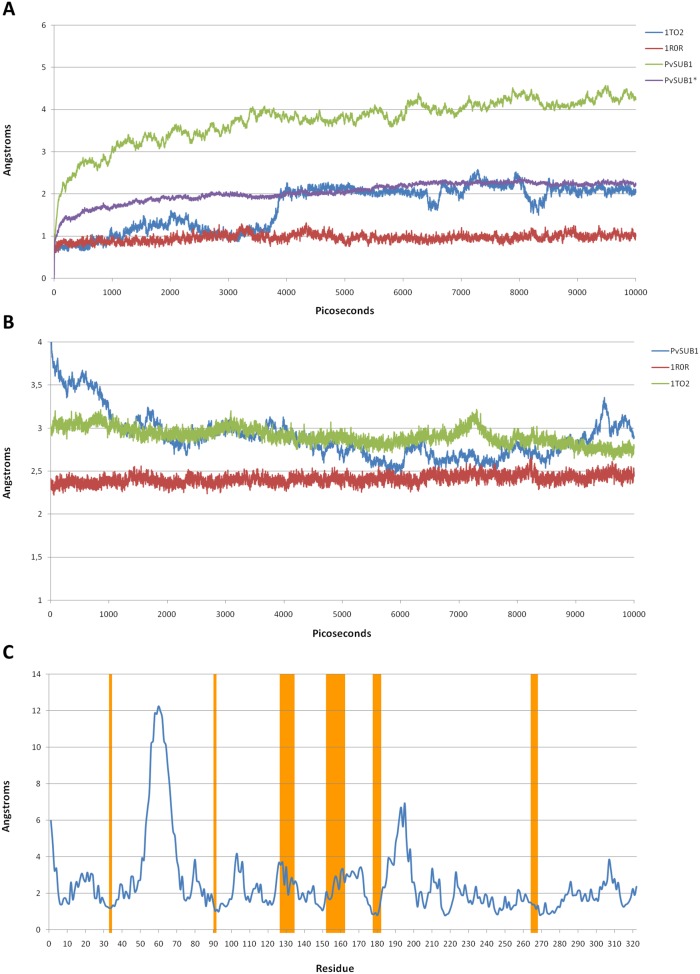
PvSUB1 molecular dynamics simulations. **A:** Average RMSD values for PvSUB1 and the 3D structure of two homologous bacterial subtilisins (1TO2, 1ROR). PvSUB1* shows the RMSD calculated without the regions missing template structural information; **B:** Fluctuation of the RMSD from the average structure. **C:** Root mean square fluctuation (RMSF) on a per-residue basis. In orange are highlighted PvSUB1 residues involved in the substrate-binding region.

Tables 1 and 2 show distance ranges among the residues of the catalytic triad, in the MD simulations of PvSUB1 and in the crystal structures of the templates. All distances fell within the ranges observed in the experimental X–ray crystal structures apart from the distances HIS372@CB-ASP316@CB and ASP316@CG-HIS372@ND1, which are slightly outside (less than 1 Å). This is consistent with the fact that this distance shows the highest variation in subtilisin X–ray crystal structures (Table 2). Stability can be also measured by analyzing the RMSD along the MD trajectories from the starting structure. In Figure 3.A, we plotted the average RMSDs obtained for the 5 MD trajectories of 10 ns each.

**Table 1:** Catalytic site distances along MD simulations.

Distance	1R0R	1T02	PvSUB1
HIS@CA-SER@CA	8.52±0.34	9.38±0.36	9.89±0.24
HIS@CB-SER@CB	6.56±0.43	7.55±0.41	7.91±0.31
HIS@CA-ASP@CA	7.78±0.52	7.91±0.29	8.33±0.18
HIS@CB-ASP@CB	6.59±0.66	6.73±0.41	7.46±0.24
SER@CA-ASP@CA	10.24±0.35	9.18±0.32	10.35±0.25
SER@CB-ASP@CB	7.42±0.42	8.83±0.49	8.72±0.34
SER@OG-HIS@NE2 *	7.54±1.11	4.69±1.19	5.11±0.71
ASP@CG-HIS@ND1	8.52±0.34	9.38±0.36	6.74±0.54
ASN@CG-SER@CB	6.70±0.71	7.5±0.91	6.52±0.41

Values are expressed in Å. The distance @@SER@OG-HIS@NE2 shows the largest fluctuation for both subtilisins with known structure and for our models, consistent with variations of this distance in subtilisin crystal structures (Table 2). Catalytic triad: Asp316, His372, Ser549; Asp137, His168, Ser325 and Asp139, His171, Ser328 for PvSUB1, 1R0R and 1TO2 respectively.

**Table 2:** Subtilisin catalytic site geometries.

Distance	lower-range	upper-range
HIS@CA-SER@CA	8.3	8.72
HIS@CB-SER@CB	6.44	6.89
HIS@CA-ASP@CA	7.22	7.46
HIS@CB-ASP@CB	5.83	6.61
SER@CA-ASP@CA	9.87	10.11
SER@CB-ASP@CB	8.15	8.53
SER@OG-HIS@NE2	2.57	3.36
ASP@CG-HIS@ND1	3.15	3.35
ASN@CG-SER@CB	6.34	6.74
SER@OH-surface	1.58	1.60

Smallest (lower-range) and longest (upper-range) measures from experimentally determined structures used as templates in the modeling of PvSUB1. The asparagine (Asn) is the residue forming the oxyanion hole. Catalytic triads of PvSUB1, bacterial subtilisins Carlsberg (1R0R) and BPN (1TO2): Asp316, His372, Ser549; Asp137, His168, Ser325 and Asp139, His171, Ser328, respectively.

The trajectories of the PvSUB1 model diverge more than those of BPN (1TO2) and Carlbserg (1R0R). This is primarily due to the regions in the model for which there is no structural information in the templates. In the model, these regions are unstructured, solvent exposed and distant from the binding pocket. When we removed them from the analysis, the RMSD reduced to values similar to those observed in the MD trajectories of subtilisin BPN (1TO2). The trend of the RMSD from the average structure shows a stabilization of the model along the MD trajectories (Figure 3.B). The per–residue fluctuation (RMSF) in Figure 3.C shows some very flexible regions, for example the region 50–80, where structural information in the templates is absent.

Most of residues forming the binding region (orange rectangles) were much less flexible than the rest, similar to what we observed for the other two subtilisins (data not shown). The fact that the model of the binding region showed similar stability in MD simulations as the X–ray crystal structures is an indication that there are no major errors in the model of this region. Obviously, despite the care we took in generating and validating our model, there may be structural errors with an effect on the success of our computational design procedure. For this reason, we included an additional step to refine the model of the structure of PvSUB1.

### Refinement of PvSUB1 model

**Figure 4:**
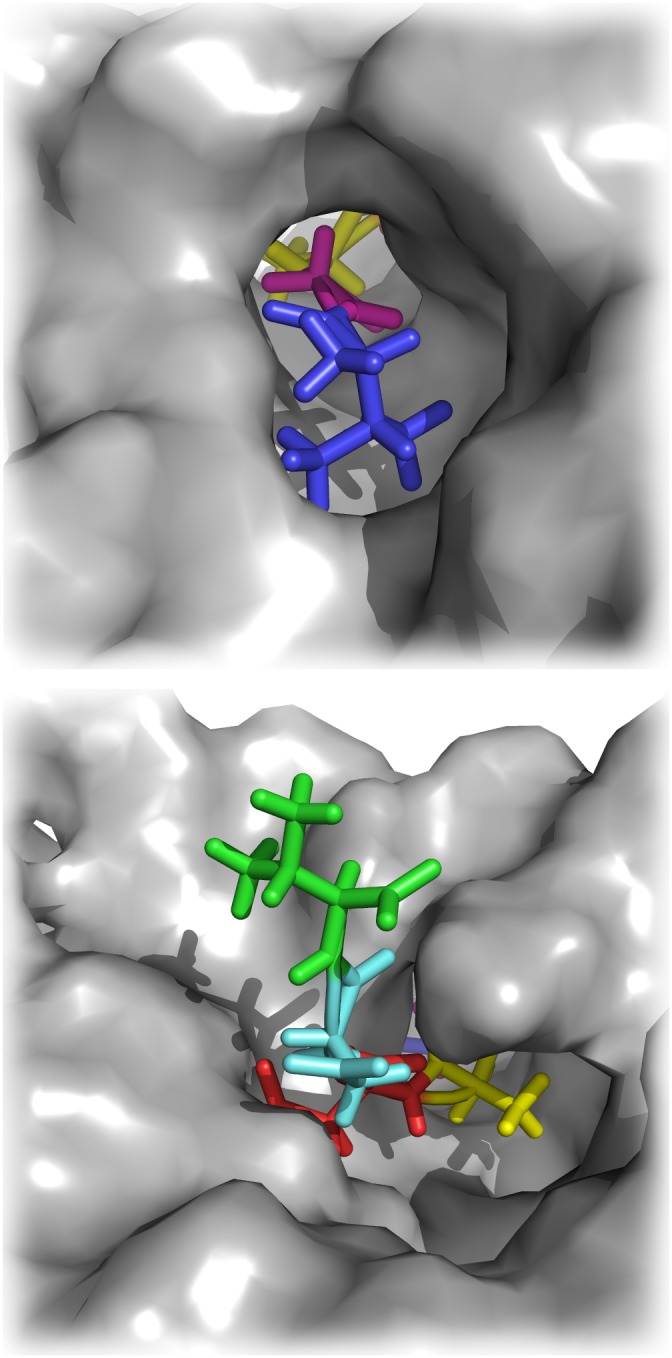
Docking of PvSUB1 hexapeptide substrate into PvSUB1 catalytic groove. Blue: P4, Violet: P3, Yellow: P2, Red: P1, Cyan: P1′, Green: P2′.

**Figure 5:**
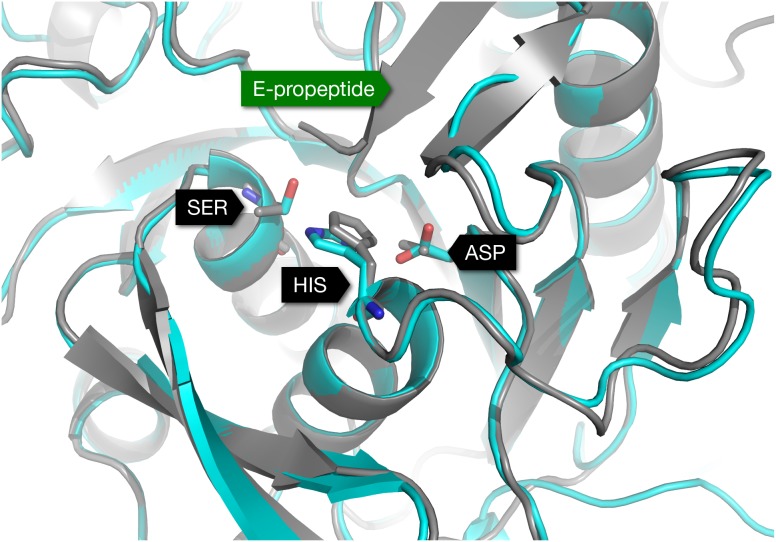
Structural alignment of the obtained PvSUB1 model (cyan) with the 3D-structure of Subtilisin E (gray, PDB 1SCJ) that was used as a template in the homology modelling. The catalytic triads in both proteins are highlighted with a stick representation. PvSUB1 catalytic triad: Asp 316, His 372 and Ser 549. Subtilisin E catalytic triad: Asp 32, His 64, Ser 221.

To obtain a refined structure of the PvSUB1 model, we performed MD simulations of the complex of PvSUB1 and its substrate hexapeptide (P4-VGADDV-P2’). The hexapeptide was docked according to the X-ray structure of Subtilisin E with its pro-peptide (1SCJ), and refined with MD, where we restrained a few distances between the protein and the peptide (see [14] for details). Even though subtilisins do not undergo major conformational changes upon binding [29], small rearrangements might take place at the interface at the level of side–chains for example. The refinement allowed us to obtain bound–like conformations of PvSUB1, which facilitated the subsequent docking step (Figure 4). Figure 5 shows the catalytic triad Ser-His-Asp of the PvSUB1 model.

### Docking of EETI-II-sub

**Figure 6:**
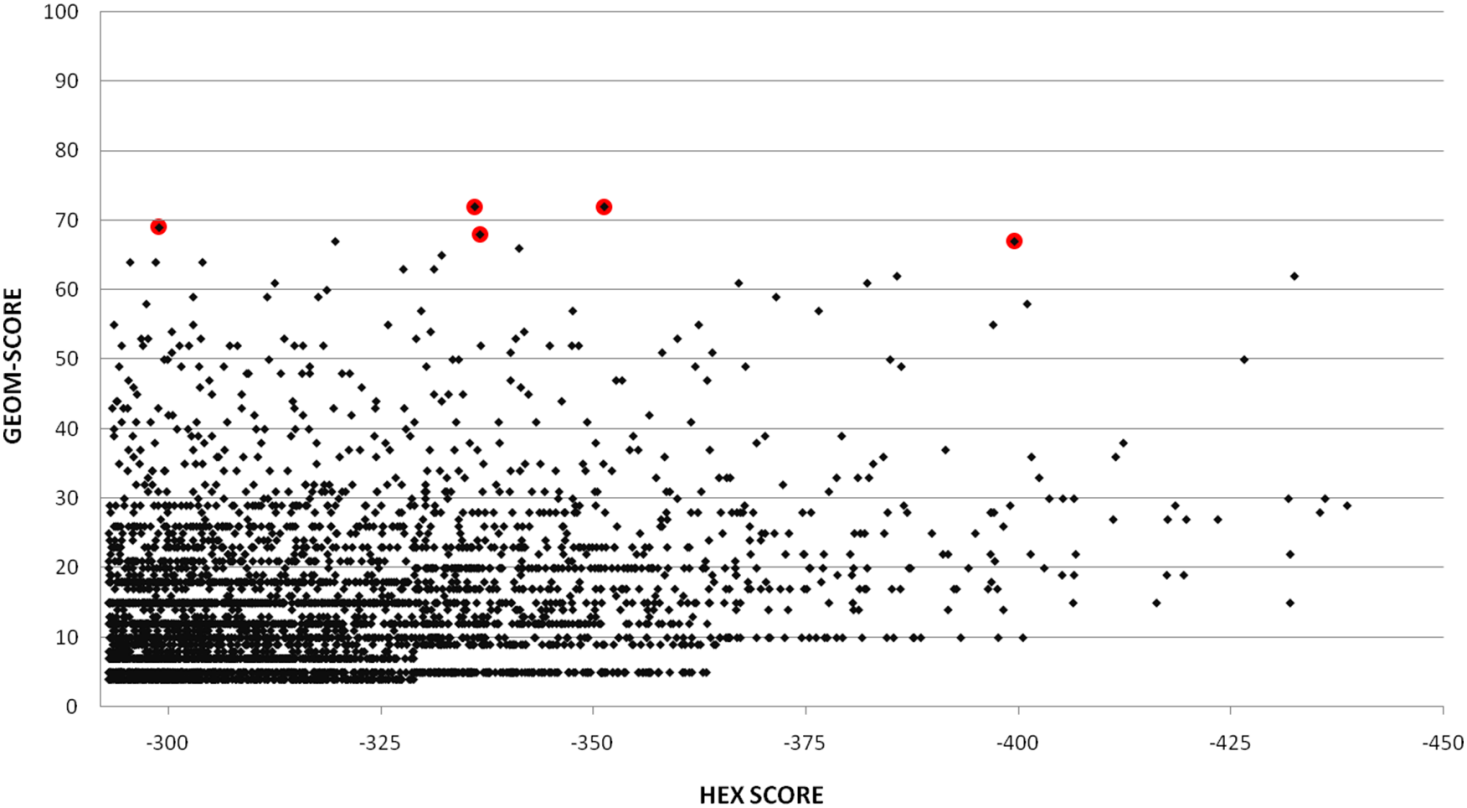
Docking results. The red circles indicate the docking poses that have been selected for refinement.

**Figure 7:**
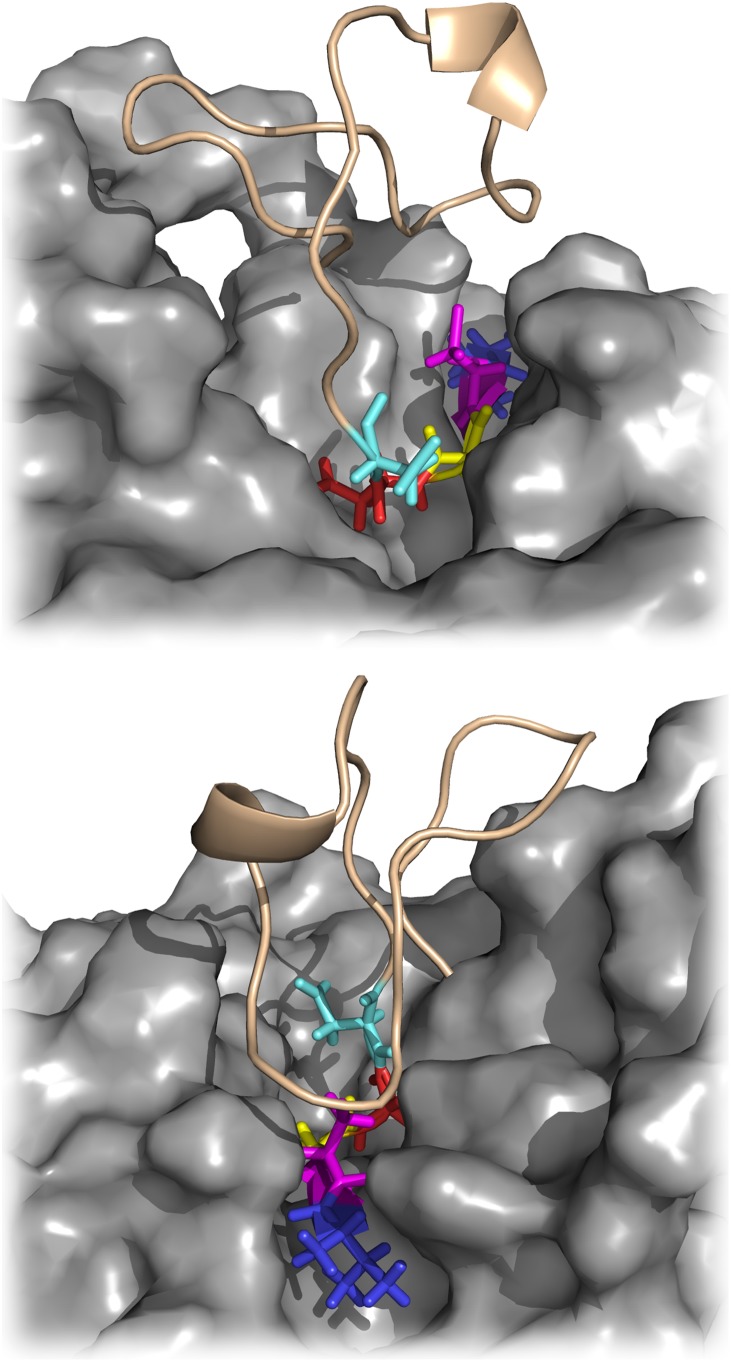
EETI-II-sub docked to PvSUB1. Blue: P4, Violet: P3, Yellow: P2, Red: P1, Cyan: P1′.

The wild type EETI-II did not inhibit PvSUB1 (Table 3). We then replaced the EETI-II residues involved in the binding to the protease catalytic groove with the sequence of the PvSUB1 natural hexapeptide substrate. This EETI-II-sub mutant inhibited PvSUB1 activity with a Ki *>*0.75 mM (Table 3). We derived snapshots (50 for each protein) for the ensemble docking of EETI-II-sub onto PvSUB1 by a cluster analysis (based on the residues at the interface) of the 5×2 ns molecular dynamics trajectories to obtain the best representative structures from the simulations. Figure 6 shows the score distribution among the solutions, with the 5 solutions with the highest geometric score (see Methods) highlighted with red circles. The relatively low geometric score indicated that most docking solutions were sub-optimal, although we had used an elaborate ensemble docking procedure. To optimize and refine the docking results we took the best 5 docking poses (red circles in 6) and refined them by a restrained molecular dynamics procedure similar to what we used for the refinement of the PvSUB1 model. This procedure helped re-establish native contacts when compared to regular unrestrained MD simulations. Subsequently we selected the solution that fulfilled all distances. Figure 7 shows the docked complex.

**Table 3:** Sequence and inhibitory activity of EETI-II mutants on PvSUB1.

Name of tested EETI-II	EETI-II active site sequences *P*_4_ *P*_3_ *P*_2_ *P*_1_ *P′*_1_ *P′*_2_	*Ki* on PvSUB1 (mM)
EETI-II-WT	G C P R I L	NI
PvS1-WT	V C A D D V	*>*0.75
PvS1-*P*_4*W*_	W C A D D V	*>*0.75
PvS1-*P*_4*P*_	P C A D D V	*>*0.75
PvS1-*P*_4*M*_	M C A D D V	*>*0.75
PvS1-*P*_4*L*_	L C A D D V	0.15±0.03
PvS1-*P*_4*I*_	I C A D D V	0.6±0.01
PvS1-*P*_4*L*_ * P*_1*E*_	L C A E D V	0.34±0.05
PvS1-*P*_4*L*_ * P*_1*K*_	L C A K D V	0.39±0.14
PvS1-*P*_4*L*_ * P*_1*R*_	L C A R D V	0.75±0.035
PvS1-*P*_4*L*_ * P*_1*Y*_	L C A Y D V	0.24±0.02
PvS1-*P*_4*L*_ * P*_1*W*_	L C A W D V	0.08±0.01

Active site sequences of the tested EETI-II mutants and their *Ki* for PvSUB1. NI: No Inhibition.

### Scoring mutants

**Figure 8:**
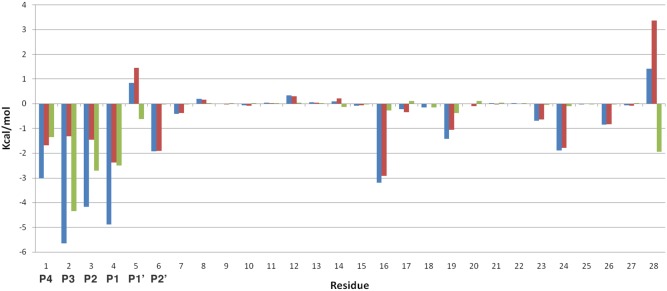
Free energy decomposition. Blue: All atoms, Red: Side chain atoms, Green: Backbone atoms. The largest contribution to the free energy of binding comes from the main-chain contacts of residues P4, P3, P2 and P1. The highest contribution comes from the cysteine in P3 and its main-chain, accounting for −4.34 kcal/mol.

A preliminary free energy calculation was performed with snapshots from multiple MD simulations of EETI-II-sub docked onto PvSUB1 to obtain more consistent MM/GBSA results [30]. A free-energy decomposition (Figure 8) shows the contribution of each single residue to the total free energy of binding.

The biggest contribution to the free energy of binding came from the main-chain contacts of residues P4, P3, P2 and P1. This is in agreement with previous observations of important interactions between a protein-inhibitor and a serine protease active site, where important contacts are made by main-chain atoms [31]. For the case of EETI-II-sub the highest contribution originates from the cysteine in P3 and its main-chain, accounting for −4.34 kcal/mol.

We then tried to identify the most favorable mutations that could improve the binding affinity of EETI- II-sub to PvSUB1. The cysteine in P3 cannot be mutated because its side-chain is involved in a disulphide bridge that has an important function in stabilizing the EETI-II scaffold and maintaining the loop rigid, whereas the alanine in P2 already contributes with −4.17 kcal/mol to the total binding energy. We also looked at the parasite sequences that are natural substrates of PvSUB1 or PfSUB1 to suggest positions to introduce mutations in the EETI-II scaffold. Table 4 lists these sequences (experimentally derived or deducted from sequence alignments) for PfSUB1 and PvSUB1. While the sequences of several PfSUB1 substrates were experimentally determined [32], [33], few were identified for PvSUB1 [15]. Considering the evolutionary proximity of *P. vivax* and *P. falciparum*, with active sites displaying *>*60% sequence identity [15], these predicted sequences can be considered reliable. Comparing the cleavage sites, we observed that only alanine and glycine appeared in P2, suggesting that only small residues are tolerated in this position. Position P1′ has a negative contribution to the energy and therefore is an interesting position to mutate. However, considering the lack of a specific pocket for this residue (Figure 7) we can consider this position almost a secondary contact residue [31] and we decided to keep the wild–type residue. Finally, SUB1 cleavage site sequences have a fairly high similarity at the P1 and P4 positions (Table 4) and we therefore focused on these positions to mutate the EETI-II inhibitory loop. It is worth mentioning that the contribution of these P1 and P4 positions within the substrate-PfSUB1 interaction has recently been experimentally established [34].

**Table 4:** SUB1 natural substrates.

*Plasmodium* protein containing a SUB1 processing site	P_4_P_3_P_2_P_1_ ↓P′_1_P′_2_	References
**Pro-region first maturation site of PfSUB1**	**VSAD** *↓* **NI**	[48]
**Pro-region second maturation site of PfSUB1**	**VSAD** *↓* **NI**	[48]
PfSERA1 site 1	IKAE*↓*AE	[6]
PfSERA2 site 1	TKGE*↓*DD	[6]
PfSERA3 site 1	VKAA*↓*SV	[6]
PfSERA4 site 1	ITAQ*↓*DD	[6]
**PfSERA5 site 1**	**IKAE** *↓* **TE**	[6] [49]
PfSERA6 site 1	VKAQ*↓*DD	[6]
PfSERA7 site 1	FKGE*↓*DE	[6]
PfSERA9 site 1	VKGS*↓*TE	[6]
PfSERA1 site 2	IYSQ*↓*ED	[6]
PfSERA2 site 2	IWGQ*↓*ET	[6]
PfSERA3 site 2	LYGQ*↓*EE	[6]
PfSERA4 site 2	VYGQ*↓*DT	[6]
**PfSERA5 site 2**	**IFGQ** *↓* **DT**	[6] [49]
PfSERA6 site 2	VHGQ*↓*SN	[6]
PfSERA7 site 2	ISAQ*↓*DE	[6]
PfSERA9 site 2	VHGQ*↓*SG	[6]
PvSERA1 site 1	TKGE*↓*DE	
PvSERA2 site 1	MKAQ*↓*DE	
PvSERA3 site 1	AKGE*↓*DE	
PvSERA4 site 1	RKAQ*↓*QQ	
PvSERA5 site 1 TKGE*↓*DE		
**PfSERA5 site 3**	VRGD*↓*TE	[6]
**PfMSP1 clone 3D7 junction MSP1**_83_ **-MSP1**_30_	**LVAA** *↓* **SE**	[50]
**PfMSP1 clone 3D7 junction MSP1**_30_ **-MSP1**_38_	**ITGT** *↓* **SS**	[50]
**PfMSP1 clone 3D7 junction MSP1**_38_ **-MSP1**_42_	**VTGE** *↓* **AI**	[50]
**PfMSP1 clone FCB1 junction MSP1**_30_ **-MSP1**_38_	**VSAN** *↓* **DD**	[51]
**PfMSP1 clone FCB1 junction MSP1**_38_ **-MSP1**_42_	**VTGE** *↓* **AV**	[51]
**PcMSP1 junction MSP1**_83_ **-MSP1**_30_	ATGE*↓*SE	[52]
**PcMSP1 junction MSP1**_30_ **-MSP1**_38_	VSAE*↓*SE	[52]
**PcMSP1 junction MSP1**_38_ **-MSP1**_42_	ANAQ*↓*ST	[52]
PvMSP1 clones Sal1 and (Belem) junction MSP1_83_ -MSP1_30_	LRGA(S)*↓*SA	
PvMSP1 clones Sal1 and Belem junction MSP1_30_ -MSP1_38_	VGGN*↓*SE	
PvMSP1 clones Sal1 and Belem junction MSP1_38_ -MSP1_42_ TTGE*↓*AE		
**PfMSP6 clone 3D7**	**VQAN** *↓* **SE**	[31]
**PfMSP7 clone 3D7 site 1**	**VKAQ** *↓* **SE**	[31]
**PfMSP7 clone 3D7 site 2**	**TQGQ** *↓* **EV**	[31]
**PfRAP-1 clone 3D7**	**IVGA** *↓* **DE**	[32]
**PfMSRP2 clone 3D7**	**LKGE** *↓* **SE**	[32]
**Pro-region first maturation site of PvSUB1**	**VGAD** *↓* **NI**	[15]
**Pro-region second maturation site of PvSUB1**	**SHAA** *↓* **SS**	[15]
**Pro-region third maturation site of PvSUB1**	**HLAG** *↓* **SK**	[15]
**Pro-region first maturation site of PbSUB1**	**VGAD** *↓* **SI**	[15]
Pro-region first maturation site of PySUB1	VGAD*↓*SI	[15]

The table shows the sequences of the cleavage sites recognized by SUB1 in in *Plasmodium falciparum* and *Plasmodium vivax*. Cleavage site sequences in bold characters have been experimentally determined, while the ones in normal characters are deducted from sequence alignments. The arrow indicates the site of cleavage between the P1 and the P1′. Cleavage site sequences have a fairly high similarity in particular at the P1, P2 and P4 positions.

**Figure 9:**
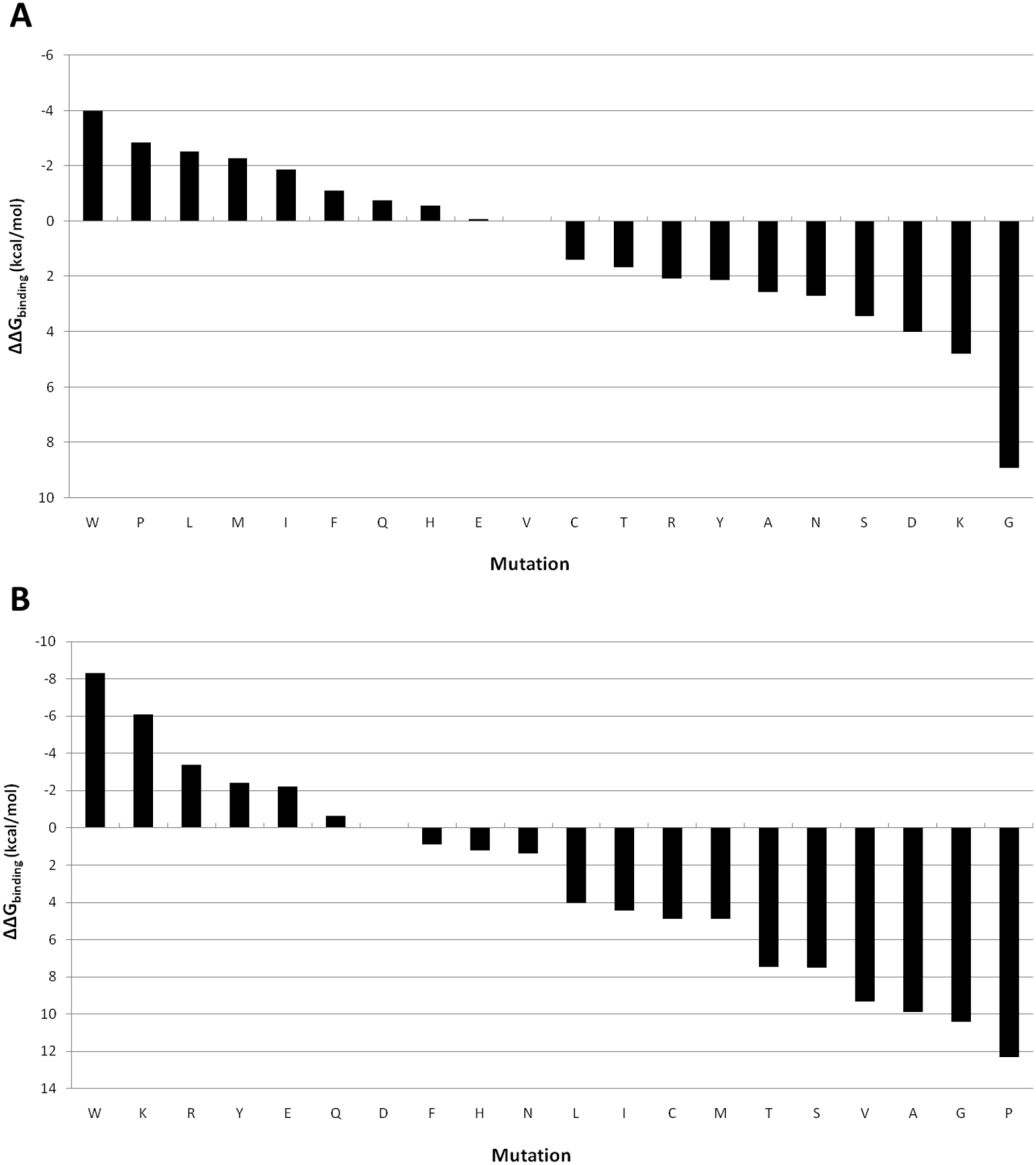
Scoring mutations on P4 and P1. **A:** mutants in position P4. The mutational profile of P4 shows that hydrophobic and bulky residues are preferred for this position. **B:** mutants in position P1. Position P1 instead prefers aromatic residues with polar groups (Tyr, Trp), glutamate and positively charged residues (Lys, Arg).

**Figure 10:**
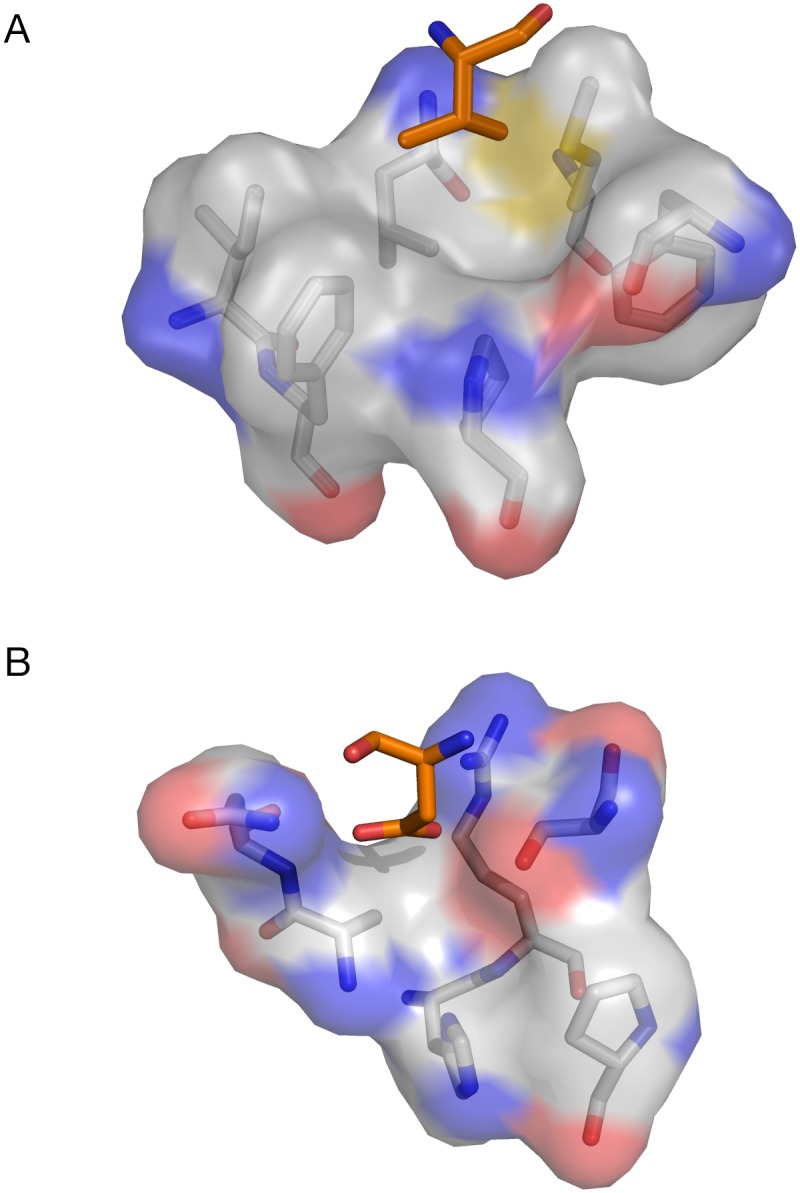
Residues forming the S1 and S4 pockets. The residue P4 (A) and P1 (B) of EETI-II are shown with an orange stick representation.

We performed 10×100 ps MD simulations and MM/GBSA free energy calculations for all possible residues in position P4 and P1 independently, assuming that the effect of the two mutations would be additive. The free energy calculations for the mutants in P4 showed that hydrophobic and bulky residues were preferred for this position (Figure 9.A). This result fits with the fact that pocket S4 is composed of six hydrophobic residues (L131, M134, F153, I161, F162, P205) and seems to have enough space to accommodate larger hydrophobic side-chains than valine (Figure 10). Position P1 instead presents as favorable mutations aromatic residues with polar groups (Tyr, Trp), glutamate and positively charged residues (Lys, Arg). Surprisingly we found as favorable mutations some positively charged residues, whereas most of sequences recognized by the homologous PfSUB1 present negatively charged (Asp, Glu) or neutral polar (Gln, Asn) side-chains at P1. This might be explained by either the low substrate specificity common to some subtilisins or imprecisions in the structure of the complex.

### Evaluation of EETI-II mutants on PvSUB1 enzymatic activity

All mutants of EETI-II were produced by chemical synthesis, folded and purified by reverse–phase HPLC. In Table 3 we present the results of PvSUB1 inhibition of the different synthesized EETI-II mutants. We initially tested mutants in position P4 according to our scoring results and found that the EETI-II with a leucine in P4 (EETI-II-P4L) inhibits PvSUB1 with a Ki of 147 µM, i.e., about one order of magnitude higher that the valine (Table 3). This could be explained by the higher flexibility of the leucine and its bulkier side-chain, which could fit more tightly into the hydrophobic S4 pocket (Figure 10). The fact that the isoleucine mutant shows a *Ki* of 591 µM, which is three times less than that of leucine, could be the effect of its beta–branched side–chain. We decided to keep the leucine mutant in P4 and test mutations in P1. Among all the EETI-II-P4L mutants for the P1 position, only one showed a better *Ki* of 86 µM, the EETI-II-P4L-P1W, which contained a tryptophane in P1. All other mutants had higher *Ki* suggesting the importance of keeping the aspartate in this position. We found the comparison of the active mutants with the list of substrate sequences of PvSUB1 in Table 4 particularly intriguing. In position P4 of the substrate there is a preference for valine and threonine, while leucine is only present in 2 out of 18 sequences; the mutant with a leucine in P4 is more than 5x more active on PvSUB1 than the one with a valine (PvS1-WT). This might be explained by the presence of important structural constraints (disulphide bonds) that are present in EETI-II compared to a more flexible conformation of the substrate sequence [29]. According to the cleavage sites predicted by sequence alignment of the substrates, we observe a prevalence of negatively charged residues such as glutamate and aspartate while there was a preference for aromatic residues in the designed inhibitors, with the tryptophan having the best *Ki*. Even in this case, the explanation might lie in the structural constraints that are present in the inhibitors and/or a set of new interactions at position P1 (Figure 10).

## Conclusions

Subtilisin-like proteins of *Plasmodium* are promising biological targets for developing novel therapeutics. One of these proteins, SUB1 plays an essential role in both the hepatic and erythrocytic stages of *Plasmodium*, making this enzyme a particularly interesting drug target. With the aim to develop an inhibitor of *Plasmodium vivax* SUB1 (PvSUB1), we redesigned a protein scaffold, the natural trypsin inhibitor EETI-II. Despite challenges in this project (the 3D structure of the target protein had to be modeled from homologues with only 30% sequence identity), the computational procedure allowed us to predict mutants that proved to be inhibitors of PvSUB1 in experimental tests.

However, some predicted good inhibitors did not show any improvements in binding to PvSUB1 when experimentally tested. This might be caused by the flexibility in the binding as proteases and their S# pockets are inherently flexible and known to alter their shape to accommodate various substrates. Imprecisions in the homology model of PvSUB1 can also have obvious consequences for the precision of the docking and of the energetic analysis. The free energy analysis based on implicit solvent methods is in itself approximate and neglects important factors influencing binding. It is encouraging that, nonetheless, we have obtained protein–based inhibitors of PvSUB1 and this opens new ways to further improve our best mutants by computational or experimental protein engineering protocols. The protein design approach described in this work demonstrates the capabilities of computational procedures to accelerate and guide the design of novel proteins with potential future therapeutic applications.

## Methods

### Modeling PvSUB1

Models of PvSUB1 were generated and validated with the same protocol as in a previous study [14]. Binding pocket residues of PvSUB1 were defined according to the interaction of canonical inhibitors with subtilisins: 152–162, 127–134, 265–268, 179–182, 90 (His), 34 (Asp), 267 (Ser). Regions were structural information from template structures is lacking are 307–324, 1–9, 51–73, 190–199, 227–231. The models were further evaluated by performing molecular dynamic simulations and comparing their dynamic behavior to that of two subtilisins 3D structures used in the homology modeling (Carlsberg: 1R0R, BPN: 1TO2). The validated model of PvSUB1 was further refined in order to obtain bound-like conformations for the ensemble docking. We docked the hexapeptide of the sequence recognized by PvSUB1 to its structure, using 1SCJ (subtilisin E + pro-peptide) as template. We performed restrained molecular dynamic simulation to refine the complex, using the protocol described in the Section “Docking Refinement”. Surface electrostatic distributions on models were calculated with the APBS [35] module implemented in Pymol.

#### EETI-II

The structure of EETI-II (2IT7) was retrieved from the PDB (http://www.rcsb.org) [36] and mutations at position P1 and P4 of the inhibitory loop were generated with the toolkit MMTSB [37]. We built the EETI-II substrate-like mutant (EETI-II-sub) by replacing its inhibitory loop (GCPRIL) with the sequence recognized by PvSUB1 (VGADDV). 3D images of the protein complexes were rendered with the molecular modeling software Pymol.

### Molecular Dynamics (MD) Simulations

All molecular dynamics simulations were performed with the SANDER module from the AMBER9 [38] package and the force–field ff99SB [39]. After minimization *in vacuo* the complexes were hydrated with TIP3P [40] water molecules and neutralized by adding an appropriate number of monovalent counterions. The MD unit cell was a truncated octahedral box with a minimum distance of 10 Å between the solute and the cell boundary. To minimize the water molecules we ran a two stages minimization protocol in which we first applied positional restraints with an energy constant of 10 kcal/(mol Å^2^) to the solute followed by a stage with 1 kcal/(mol Å^2^) energy constant. Both minimization stages consisted of 10 steepest-descent and 490 conjugate-gradient steps. During the equilibration/heating and production dynamics all covalent bonds to hydrogen atoms were constrained with the SHAKE [41] algorithm, and we used a time step of 2 fs. We used periodic boundary conditions with a distance cutoff of 8.0 Å for the direct part of the non-bonded interaction and PME [42] (Particle Mesh Ewald) to account for long-range electrostatic interactions. The minimized system was then thermalized and equilibrated by heating from 0 to 300°K over 20 ps under constant-volume conditions followed by 10 ps at constant-pressure. The production MD phase was launched from the final configuration after equilibration with a relaxation time of 2.0 ps for heat bath coupling and a pressure relaxation time of 2.0 ps.

### Docking

The pool of conformers used for the ensemble docking was obtained from multiple (5×2 ns) MD trajectories of PvSUB1 (receptor) and EETI-II (ligand). We extracted snapshots every 10 ps of MD for a total of 1000 snapshots of each protein and clustered these structures with a single-linkage algorithm implemented in GROMACS [43] (g-cluster tool), where the RMSD was calculated only for the binding interface residues. We selected for cross-docking the centroids of each of the 50 clusters identified. The ensemble docking was performed with the rigid-body docking software HEX [44] (version 4.5) where the search was restricted to the binding pocket by positioning EETI-II structures around the interface and limiting the search to 30° for twist range, receptor and ligand range. We used a shape + electrostatic correlation type while the other parameters of HEX were left as default. The best 5000 docking solutions according to the HEX score were selected and re-ranked by a mixed score based on the geometry of the interaction between a canonical loop inhibitor and a subtilisin. This score (geometric score) was composed for 40% of the HEX score (based on surface complementarity) and for 60% of an empirical score defined by the conserved distances between atoms in the inhibitory loop of a canonical inhibitor and a subtilisin. A mixed score permits to take into consideration the shape complementary and the conserved structural feature of the interaction. The best 5 solutions were selected for refinement.

### Docking Refinement

The refinement is based on a protocol that uses restrained molecular dynamics simulations. The refinement procedure consisted of a total of 360.000 steps based on specific distance restraints at the ligand/receptor interface and used the NMR refinement tools in AMBER9. The chosen conserved distance restraints were 267SER@OG-P1@C (lower bounds = 2.5 Å, lower-intermediate = 3.0 Å, intermediate- upper = 4.0 Å, upper bounds = 5.5 Å), 154SER@HN-P3@O, 154SER@O-P3@HN, 129GLY@HN-P4@O, 129GLY@O-P4@HN, 127LYS@O-P2@HN (lower bounds = 1.5 Å, lower-intermediate = 2.0 Å, intermediate- upper = 3.0 Å, upper bounds = 3.5 Å). In all cases, an energy constant of 20 kcal/mol Å^2^ was employed. The refinement consisted of three phases. In the first phase (120.000 steps), the receptor (PvSUB1) was kept rigid by applying a Cartesian restraint with an energy constant of 10 kcal/(mol Å^2^) and the distance restraints were switched on/off every 15.000 steps. In the second phase a Cartesian restraint with an energy constant of 10 kcal/(mol Å^2^) was applied only to heavy main-chain atoms keeping side-chains fully flexible, and in the third phase the energy constant of this Cartesian restraint was reduced to 0.01 kcal/(mol Å^2^). During these phases, the ligand (EETI-II or hexapeptide) was kept completely flexible. An additional 1 ns of regular MD simulation was performed to allow the system to relax into its final configuration. In the validation, this resulted in a high accuracy complex structure, with no distortions at the interface.

### Scoring

The MM/GBSA protocol [45] in AMBER9 was used to calculate the relative free energy of each mutant. The default GBSA model used in the calculations was that of Tsui and Case [46] with an external dielectric of 80 and internal dielectric of 1.0. For calculating the nonpolar contribution, the surface tension coefficient was set to 0.0072 and the surface offset to 0.0. The solvent accessible surface area was calculated with the ICOSA method. We calculated the relative free energy of binding from snapshots extracted each 10 ps from 10×100 ps trajectories.

### Protein Production

#### EETI-II and mutants

All peptides (desalted, 35%–60% pure as assessed by HPLC) were obtained from GenScript Corporation, Piscatway, NJ, USA. In a typical procedure, peptide (50 mg) was dissolved in 75 mL of KH_2_PO_4_ buffer (0.2 M, pH 8.2) and allowed to air-oxidize at room temperature under gentle stirring. Monitoring was achieved with Ellman’s test [47] and analytical HPLC (column ACE C18, 5 µm×4.6 mm, eluent A: 0.1% TFA/H_2_O, eluent B: 60% CH_3_CN/H_2_O/0.1% TFA) with a 30 min linear gradient of 25% to 55% B at 1 mL flow rate (monitoring at 210 nm). When Ellman’s tests are negative and the HPLC monitoring shows no more trace of starting materials (after 3 to 5 days), the reaction mixture was centrifuged and the supernatant loaded onto a preparative HPLC column (Merck Lichrospher C_18_, 10 µm, 250×25 mm). Elution was achieved with a 90 min linear gradient of 25% to 55% B at 10 mL flow rate (monitoring at 220 nm). The fractions containing the oxidized peptide were combined and lyophilized to yield 15 to 30% of the desired peptide. Successful oxidation was confirmed by mass spectrometry (MALDI-Tof, Bruker Biflex III).

#### Production and purification of the PvSUB1 recombinant enzyme

The production and purification of the PvSUB1 (Genbank accession number FJ536585) recombinant enzyme was performed essentially as previously described [14], [15]. Briefly for large-scale protein production, *Spodoptera frugiperda* Sf9 insect cells (1L at 3×106 cells/mL, Invitrogen) were infected for 72 h with recombinant PvSUB1-recombinant baculovirus at a Multiplicity Of Infection (MOI) of 10 in Insect XPRESS medium (Lonza) supplemented with 50 µg/mL gentamycin and 0.5 µg/mL tunicamycin (Sigma-Aldrich). Culture supernatant containing the secreted PvSUB1 recombinant protein was harvested, centrifuged 30 min at 2150 g to remove cells and cellular debris and concentrated/diafiltrated against D-PBS 0.5 M NaCl, 5 mM Imidazole (loading buffer). The proteins were purified on an AKTA purifier system (GE Healthcare). The sample was loaded onto a 3 mL TALON Metal affinity resin (Clontech Laboratories) equilibrated in loading buffer. After extensive washes with loading buffer, the bound protein was eluted with a linear gradient of 5–200 mM imidazole in D-PBS, 0.5 M NaCl. Fractions containing PvSUB1 were pooled and concentrated by using Amicon Ultra 15 (10000 MWCO) and size-fractionated onto a HiLoad 16/60 Superdex 75 column equilibrated with 20 mM Tris pH 7.5, 100 mM NaCl. Fractions were monitored by absorbance (280 nm) and analyzed by Coomassie blue staining of SDS-PAGE gels and enzyme activity assay. The fractions containing the recombinant enzyme activity were pooled and the protein concentration was determined using the BCA Protein Assay following manufacturers recommendations (Bio Basic). Purified PvSUB1 recombinant protein was stored at −20°C following the addition of 30% v/v of pure Glycerol.

### Enzymatic Test

For the kinetic assays we used the purified recombinant PvSUB1 enzyme and its specific peptide sub- strate whose sequence is deduced from PvSUB1 auto-maturation site: KLVGADDVSLA, with cleavage occurs between the two aspartates for PvSUB1. The KLVGADDVSLA sequence was coupled to the fluorophore/quencher dyes Dabsyl/Edans (Exc/Em 360/500 nm) at each edge. The enzymatic assays were performed in 20 mM Tris pH 7.5 and 25 mM CaCl_2_ at 37°C as previously described [15]. For the determination of the Ki, the compounds, previously resuspended in ultra-pure distilled water at 10 mM, were tested at ten different concentrations ranging from 1 mM to 2 µM following sequential 1∶2 dilutions. The final mixture was distributed in duplicate into a 384-well black microtiter plate (Thermo Scientific) and the fluorescence was monitored every 3 minutes for 90 min at 37°C in a Labsystems Fluoroskan Ascent spectro-fluorometer. The slope of the linear part of the kinetic was determined in an Excel (Microsoft) spreadsheet. Every steps of the enzymatic assay were done on ice to make sure that the protein was not active before the measure of the fluorescence. The Ki and IC50 values were determined (N = 3) using GraphPad Prism software.
